# Evolution of Gas Exchange Abnormalities in Patients with Liver Cirrhosis Candidate for Liver Transplantation

**Published:** 2012-03-01

**Authors:** S M A Ghayumi, S Mehrabi, M Hoseini Asl

**Affiliations:** 1 Department of Internal Medicine, Nemazee Hospital, Shiraz University of Medical Sciences, Shiraz, Iran

**Keywords:** Blood gas, Cirrhosis, Hypoxemia

## Abstract

**Background:**

Hypoxemia is common in patients with cirrhosis but the natural history of this syndrome is unknown. This study was conducted to evaluate the natural history of arterial oxygenation in patient with end stage liver cirrhosis.

**Methods:**

Sixty eight patients with liver cirrhosis were followed up for 6-12 months. Arterial blood gas (ABG) and pulse oximetry were obtained on day of presentation and follow up.

**Results:**

There were no significant changes in the oxygen saturation by pulse oximetry (SpO2), partial pressure of oxygen (PaO2) and alveolar arterial oxygen gradient (A-a O2) after 6-12 months. Mean arterial oxygen saturation (SaO2) in 46 patients was 95.42±1.92, and after follow up changed to 95.45±2.96. Thirty eight patients had SaO2 > 94% (mean 96.12±1.08 after 6-12 months changed to 95.66±2.58) ; 8 patients had SaO2 = 94 (mean 92.08±1.44 after 6-12 months changed to 94.46±4.47).

**Conclusion:**

There were no significant changes in the SpO2, PaO2 and A-a O2 after 6-12 months.

## Introduction

Abnormalities of pulmonary gas exchange are common in patients with liver cirrhosis.[1] The prevalence of hypoxemia reported in the literature varies from about 22% to 70% in the different patient populations[2][3] and widened A-a oxygen gradient in up to 69% of patients.[1] It may be partly caused by concomitant lung or cardiac dysfunction, but the liver disease per se and its complications may also influence these gas exchange abnormalities.[2][4][5]

The natural history of patients with cirrhosis and hypoxemia and their evolution are unknown.[6] Since chronic liver diseases usually progress gradually, one suspects that oxygenation also may be impaired gradually.[3] Swanson et al. recommended repeated arterial blood gas studies within 12 months while on the orthotic liver transplantation (OLT) waiting list.[7]

In the current study, we determined the changes of gas exchange abnormalities in a series of patients with end stage liver disease candidate for liver transplantation.

## Materials and Methods

Sixty eight patients were followed up for a period of 6-12 months. Six patients were in Child A, 38 in Child B and 24 in Child C. Cirrhosis was proven histologically in all patients. Forty seven patients were male with mean age of 41.87±12.13 years, 21 female (34.81±14.15). Seventeen patients were followed up for 12 months. Spirometry and Doppler echocardiography were obtained in all patients. FVC was > 80% in 57 patients and ABG were obtained in 46 patients at least on two occasions. Hypoxemia was defined as A-a O2 ≥ 20 mmHg.[6]

Arterial blood gas (ABG) was obtained in the sitting position while breathing room air with standard technique from radial artery and included oxyhemoglobin saturation (SaO2), arterial O2 pressure (PaO2), pH, and bicarbonate (HCO3) level. Oxygen saturation by pulse oximetry (SpO2) was determined by a finger pulse oximeter device (Oxypleth® 520A pulse oximeter, Novametrics, Respironics Inc., Murrysville, PA) on patients' right forefinger in room air after remaining in sitting position for 15 minutes and the stable value after 1 minute of pulse oximetry defined as SpO2.

SPSS for windows (SPSS Inc., Chicago, IL) version 15 was used for statistical analysis. Values were expressed as the mean±SD. Group differences were assessed using Student's paired t test for 35 and more and Wilcoxon test for less than 35 patients. Differences in categorical variables between groups were assessed by the Chi-Square test or Fisher's Exact test when appropriate. All tests were two-sided. Values of p <0.05 were considered to indicate statistical significance.

## Results

A total of 68 patients with liver cirrhosis, 47 males (69%) and 21 females (31%) were followed at our clinic as outpatients were included in this study and followed for 6-12 months. The mean age of patients was 39.69±13.10 years. Seventeen patients were followed for 12 months. The PaO2 worsened in 25 patients and improved in 21 patients. There were no significant changes in the SpO2, PaO2 and A-a O2 after 6-12 months of follow up. Table 1 shows gas exchange abnormalities before and after 6-12 months of follow up.

**Table 1:** Pulmonary gas exchange findings before and after six months in patients with cirrhosis.

		**Number of cases **	**Time 0 **	**After 6-12 month **	***P* value **
PaO_2_		46	79.59±9.90	81.11±12.84	0.440
	>70	38	82.88±1.15	82.53±7.09	0.862
	≤70	8	63.93±5.06	74.34±14.96	0.546
SaO_2_		46	95.42±1.92	95.45±2.96	0.953
	>94%	38	96.12±1.08	95.66±2.58	0.306
	≤94	8	92.08±1.44	94.46±4.47	0.165
SpO_2_		68	96.84±2.45	96.91±2.18	0.710
	>94%	64	97.31±1.04	97.21±1.22	1.00
	≤94	4	89.25±5.50	87±4.24	0.500
A-a O_2_		46	22.89±15.34	21.09±14.41	0.386
	<20	23	12.99±5.37	9.85±5.97	0.002
	≥20	23	29.26±6.97	29.33±9.22	0.005

Twenty three patients had A-a O2 = 20 mmHg, and changes of A-a O2 were significant in this group of patients after follow up (29.26±6.97 changed to 29.33±9.22) (p=0.005). There was no significant association between hypoxemia and Child Pugh classification (Table 2).

**Table 2:** Evolution of oxygenation according to child score.

**Child score (number of cases)**	****	**Start of Study**	**After 6 months**	***P* value**
Child A (6)	PaO2	77.40±4.07	82.37±12.60	0.465
	SaO2	95.73±0.98	93.78±4.66	0.428
	SpO2	97.80±0.045	97.20±80.04	0.083
Child B (38)	PaO2	84.60±23.48	84.00±15.89	0.500
	SaO2	95.37±3.27	95.97±2.30	0.143
	SpO2	96.73±2.31	96.97±2.41	0.103
Child C (24)	PaO2	76.50±10 04	77.29±17 68	0.777
	SaO2	94.36±3.73	91.73±12.20	0.391
	SpO2	96.65±2.93	96.61±2.04	0.835

Twenty eight patients had abnormal spirometric results (restrictive) without any significant association with hypoxemia. Three patients had SaO2 = 90% mean=84.47±4.76, after 6 month changed to 89.90±4.97 (p=0.019); 51 patients had SaO2 > 90% mean 95.70±1.80 after 6 month 94.58±7.60 (p= 0.31). Six patients had PaO2 < 65, mean 54.83±9.22, after 6 month 62.30±13.14 (p=0.201).

## Discussion

This study shows that more than 50% of patients with cirrhosis had hypoxemia which confirm previous clinical investigations that hypoxemia is common in patients with cirrhosis.[6][8][9]

Moller et al. in a study2 found that severe hypoxemia and increased alveolar-arterial oxygen gradient were associated with the severity of the liver disease. In another study,[6] the evolution of the A-aO2 was unpredictable and not related to changes in Child–Pugh score in patients with hypoxemia or in those without hypoxemia. On the other hand, Swanson et al. in their study showed deterioration of oxygenation while awaiting OLT in 12 of 14 hepatopulmonary syndrome (HPS) patients (86%) within a 12-month period. The rate of decline in PaO2 was variable but was clinically significant.

In contrast with Aller,[10] our study shows the lack of correlation between the degree of hypoxemia and pulmonary function tests and Child-Pugh score, suggesting that other factors play a role in the occurrence of the hypoxemia in patients with cirrhosis.[11] Also in our study, the evolution of the A-aO2 was unpredictable in patients with hypoxemia or in those without hypoxemia.

Although this study was performed only for a short period, the A-aO2 values changed in most patients, suggesting that this variable was unstable. In contrast with previous studies,[4][12][13] certain patients with hypoxemia normalized their A-aO2 gradient, suggesting that hypoxemia may be transient in patients with severe cirrhosis. The results of this study in contrast with Vachiry showed no association between hypoxemia and severity of cirrhosis.[14]

The findings of our study demonstrated no changes in PaO2, SaO2, SpO2 and A-a O2 in 12 months follow up of patients with end-stage liver disease and these findings were also seen in the subgroup of hypoxemic patients ( PaO2 < 65 mmHg).
